# Intensive Periodontal Treatment Reduces Risks of Hospitalization for Cardiovascular Disease and All-Cause Mortality in the Hemodialysis Population

**DOI:** 10.3390/jcm7100344

**Published:** 2018-10-11

**Authors:** Shih-Ting Huang, Tung-Min Yu, Tai-Yuan Ke, Ming-Ju Wu, Ya-Wen Chuang, Chi-Yuan Li, Chih-Wei Chiu, Cheng-Li Lin, Wen-Miin Liang, Tzu-Chieh Chou, Chia-Hung Kao

**Affiliations:** 1 Division of Nephrology, Taichung Veterans General Hospital, Taichung 407, Taiwan; kitheroborn@hotmail.com (S.-T.H.); yu5523@gmail.com (T.-M.Y.); wmj530@gmail.com (M.-J.W.); colaladr@yahoo.com.tw (Y.-W.C.); 2 Graduate Institute of Public Health, China Medical University, Taichung 404, Taiwan; 3 Graduate Institute of Biomedical Sciences and School of Medicine, College of Medicine, China Medical University, Taichung 402, Taiwan; 4 Division of Nephrology, Ministry of Health and Welfare Chiayi Hospital, Chiayi 600, Taiwan; nightrider731@msn.com; 5 Graduate Institute of Clinical Medical Science, China Medical University, Taichung 404, Taiwan; D17550@mail.cmuh.org.tw (C.-Y.L.); givychiu@gmail.com (C.-W.C.); 6 Department of Anesthesiology, China Medical University Hospital, Taichung 404, Taiwan; 7 Management Office for Health Data, China Medical University Hospital, Taichung 404, Taiwan; orangechengli@gmail.com; 8 College of Medicine, China Medical University, Taichung 404, Taiwan; 9 Graduate Institute of Biostatistics, China Medical University, Taichung 404, Taiwan; wmliang@mail.cmu.edu.tw; 10 Department of Public Health, China Medical University, Taichung 404, Taiwan; 11 Department of Health Risk Management, College of Public Health, China Medical University, Taichung 404, Taiwan; 12 Department of Nuclear Medicine and PET Center, China Medical University, Taichung 404, Taiwan; 13 Department of Bioinformatics and Medical Engineering, Asia University, Taichung 404, Taiwan

**Keywords:** cardiovascular disease, end-stage renal disease, hemodialysis, periodontal disease, stroke

## Abstract

Periodontal disease (POD) is associated with the risk of atherosclerotic vascular disease in patients on hemodialysis (HD). The association between POD treatment and cardiovascular diseases (CVDs) is still unknown. A total of 3613 patients who received HD and intensive POD treatment between 1 January 1998, and 31 December 2011 were identified from the National Health Insurance Research Database as the treatment cohort. The comparison cohort comprised patients without POD treatment who were matched to the patients in the treatment cohort at a 1:1 ratio by the propensity score. All CVDs defined by International Classification of Diseases, Ninth Revision (International Classification of Diseases, Ninth Revision (ICD-9)) codes were ascertained by hospital records for nonfatal events. The first CVD was used to define incidence. Relative risks were estimated by hazard ratios from the Cox proportional hazard model with adjustment for demographic variables and cardiovascular risk factors. Compared with the comparison cohort, the adjusted hazard ratio of hospitalization for CVDs was 0.78 (95% confidence interval = 0.73–0.84, *p* < 0.001) in the treatment cohort The treatment cohort exhibited significantly lower cumulative incidences of CVDs (log-rank test *p* < 0.001) and mortality (log-rank test *p* < 0.001). Intensive POD treatment was associated with reduced risks of CVDs and overall mortality in patients on HD.

## 1. Introduction

Cardiovascular disease (CVD) accounts for approximately 50% of deaths in patients on dialysis. The increased incidence of CVD in patients undergoing dialysis is attributed to increased prevalence of traditional risk factors for CVD such as diabetes mellitus (DM), dyslipidemia, hypertension [1,2], and risk factors due to loss of renal function [3]. Malnutrition–inflammation–atherosclerosis syndrome and hemodynamic induced vasculopathy/cardiomyopathy are additional risk factors from inadvertent effect of renal replacement therapy in dialysis patients and play central roles in cardiovascular complications [4,5].

Therapeutic interventions have been taken for secondary prevention and reduction of mortality in patients on dialysis. These include adequate dialysis [6]; measures to reduce recovery time after dialysis sessions [7]; adequate nutrition [8]; anemia treatment [9]; management of diabetes, hypertension, and dyslipidemia [10]; and the use of phosphate binders [11]. The use of statins for CVD management in patients on dialysis is still controversial [12]. Although the number of cardiovascular deaths has declined in the general population, a similar trend has not been observed for patients on dialysis despite the implementation of the modern therapy modality and pharmacological therapy [5]. In addition, no pharmacologic agents are recommended to reduce chronic inflammation and modify outcomes in patients with kidney disease. However, inflammation resulting from an ongoing occult infection should be aggressively treated.

Approximately 80.6% of patients on dialysis have periodontal disease (POD) [13]. The pathogenesis of periodontitis in patients with end-stage renal disease (ESRD) includes reduced salivary production, increased salivary urea levels, elevated parathyroid hormone levels, and malnutrition [14]. Periodontitis is correlated with chronic inflammation and malnutrition in patients on hemodialysis (HD) [13]. Periodontal health is also correlated with markers of malnutrition and inflammation in the dialysis population [14].

Evidence indicates that POD is associated with increased all-cause and cardiovascular mortality in the HD population [15,16]. Periodontal therapy has been reported to improve systemic inflammation, nutritional status, and erythropoietin responsiveness in the HD population [17]. However, the clinical effects of intensive POD therapy on cardiovascular prevention in patients on dialysis remain unclear. Our study investigated the effect of intensive POD therapy on cardiovascular risks and examined the trends of outcome risks modified by the treatment responsiveness of periodontal therapy.

## 2. Methods

### 2.1. Data Source

This retrospective, observational cohort study examined data from the Registry of Catastrophic Illness Database (RCID), a subset of the National Health Insurance Research Database (NHIRD). The Taiwan Bureau of National Health Insurance established the universal National Health Insurance (NHI) program in 1995, which currently provides comprehensive medical coverage to 99% of the population of Taiwan (23.72 million residents) (https://www.nhi.gov.tw/english). The National Health Research Institutes (NHRI) maintain the NHIRD for research purposes. The RCID includes data for all patients who fulfill the guidelines of the National Health Insurance Administration for a catastrophic illness certificate. Catastrophic illnesses are defined as severe conditions requiring advanced health care, such as malignancies, post transplantation status, and ESRD. Patients with a catastrophic illness certificate are exempted from making any copayment for medical care, and this certificate is formally reviewed by physicians at the time of application. The NHIRD identifies diseases based on the International Classification of Diseases, Ninth Revision, Clinical Modification (International Classification of Diseases, Ninth Revision, Clinical Modification (ICD-9-CM)). The accuracy and validity of NHIRD diagnostic codes have been established [18,19].

### 2.2. Ethics Statement

The NHIRD encrypts patient personal information to protect privacy and provides researchers with anonymous identification numbers associated with relevant claims information, including sex, date of birth, medical services received, and prescriptions. Therefore, patient consent is not required to access the NHIRD. This study was approved to fulfill the conditions for exemption by the Institutional Review Board (IRB) of China Medical University (CMUH-104-REC2-115R3). The IRB also specifically waived the consent requirement.

### 2.3. Sampled Patients

**Figure 1:**
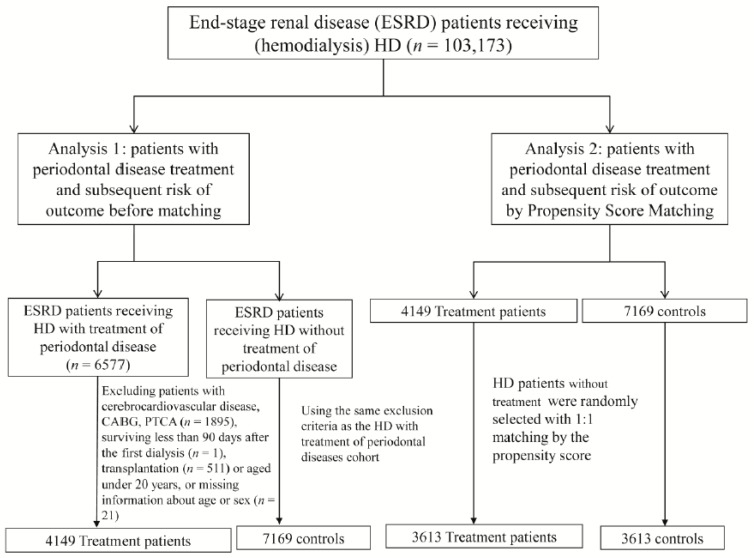
Patient selection flow chart. ESRD, end-stage renal disease; HD, hemodialysis; CABG, coronary artery bypass graft; PTCA, percutaneous transluminal coronary angioplasty.

Figure 1 presents a flow chart for selecting patients for the study groups. Patients newly diagnosed with ESRD (ICD-9-CM 585) between 1 January 1998 and 31 December 2011 and those who underwent dialysis for at least 3 months were identified from the RCID. Patients were excluded if they had acute coronary syndrome (ACS; ICD-9-CM codes 410, 411.1, and 411.8), stroke (ICD-9-CM codes 430–438), or congestive heart failure (CHF; ICD-9-CM code 428) before enrollment; survived for fewer than 90 days after the first dialysis date; were undergoing transplantation; were aged under 20 years; or had missing demographic information. Periodontal treatments provided in this study were subgingival curettage (scaling (91004C) and root planning (91006C, 91007C, and 91008C)) and periodontal flap surgery (91009B and 91010B) (*n* = 4149). For each periodontal treatment patient identified, controls free of periodontal treatments were randomly selected from the same period according to the same exclusion criteria.

**Figure 2:**
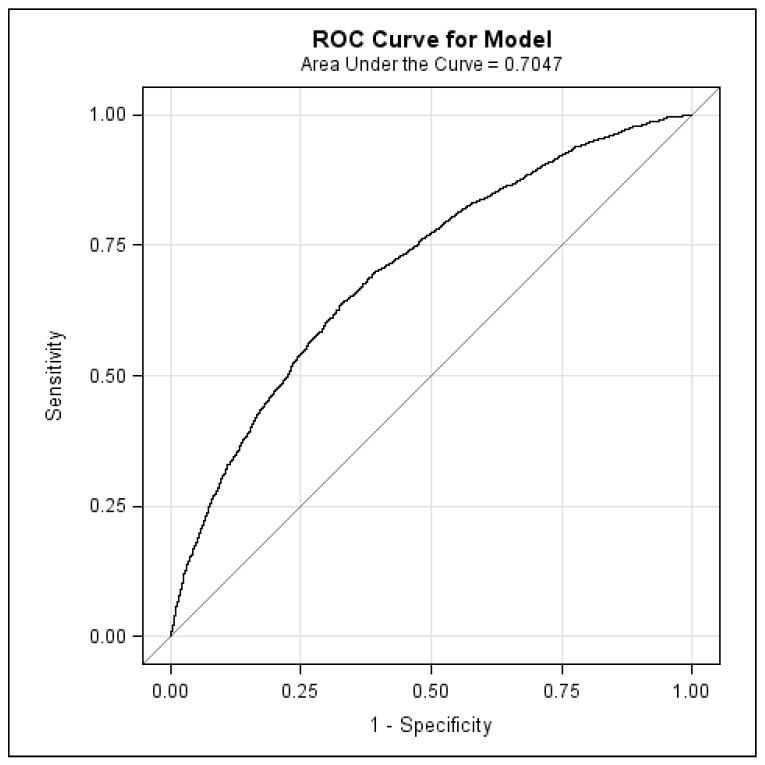
The receiver operating characteristic cure (ROC) for the logistic regression for propensity score. The C-statistic (area under curve, AUC) was 0.70.

We further established a comparison cohort comprising patients without POD who were matched to the treatment cohort at a 1:1 ratio by the propensity score [20]. The propensity score was calculated using logistic regression to estimate the probability of treatment assignment according to the baseline variables of age, sex, dialysis duration, urbanization level, monthly income (in New Taiwan Dollars (NTDs)), medication use, comorbidities, and Charlson comorbidity index. The C-statistic of the logistic regression model was 0.70 (Figure 2). The index date was defined as either the date of POD therapy for the treatment cohort or the 15th day of the same month for the comparison cohort.

### 2.4. Outcome Measurement

All individuals were followed up until a new outcome diagnosis based on the hospitalization records. Individuals were censored at the date of death, or on loss to follow-up, withdrawal from the insurance system, or on 31 December 2011, whichever came first. The primary endpoint was all-cause mortality. The identification of death events was based on hospital discharge because of death and withdrawal from the NHI, as indicated in the NHIRD.

The secondary endpoints were hospitalization for newly diagnosed CVDs, ACS, acute myocardial infarction (AMI; ICD-9-CM code 410), ischemic stroke (ICD-9-CM codes 430–436 and 437.1), hemorrhagic stroke (ICD-9-CM codes 431, 767.0, and 772.2), CHF, and sudden cardiac death (SCD; ICD-9-CM codes 427.5, 798.1, and 798.2).

### 2.5. Independent Variables

Sociodemographic and comorbidity data with age, sex, urbanization level, and monthly income as the covariates were obtained from the claims data. The criteria published by the NHRI on urbanization levels in Taiwan were adopted. The criteria consist of seven strata; level 1 denotes the highest urbanization level, and level 7 denotes the least urbanized communities. The standards for classification were the population density (people/km^2^); population ratios of elderly people, agricultural workers, and people with different educational levels; and the number of physicians per 100,000 people. The urbanization level was divided into two groups based on an NHRI report: levels 1 and 2 denoted cities and levels 3–7 denoted rural areas. The monthly costs of insurance premiums incurred by patients were classified into three groups: <NTD15,000, NT$15,000–19,999, and ≥NTD20,000 (USD1 is equivalent to approximately NTD30). Comorbidities diagnosed before the index date included chronic obstructive pulmonary disease (COPD; ICD-9-CM codes 491, 492, and 496), hyperlipidemia (ICD-9-CM code 272), diabetes (ICD-9-CM code 250), hypertension (ICD-9-CM codes 401-405), peripheral arterial occlusive disease (ICD-9-CM code 433.9), cancer (ICD-9-CM codes 140-239), and lower extremity amputation (ICD-9-CM code V49.70).

### 2.6. Data Availability Statement

All data and related metadata were deposited in an appropriate public repository. The data on the study population that were obtained from the NHIRD (http://nhird.nhri.org.tw/en). The NHRI is a nonprofit foundation established by the government.

### 2.7. Statistical Analysis

The demographic characteristics and prevalence of comorbidities were compared between the HD cohort without POD treatment and the HD cohort with POD treatment. Baseline characteristics of the before-matched cohorts and propensity score-matched cohorts were compared using standardized mean differences. A value of standardized mean differences equals 0.05 or less, which indicates a negligible difference in means. The incidence density of each disease outcome per 1000 person-years was calculated for each cohort. Multivariable Cox proportional hazards models were used to estimate the hazard ratios (HRs) and 95% confidence intervals (CIs) of outcomes and were adjusted for variables that were significantly related to outcomes from the prior univariate Cox regression model. Furthermore, we considered death a competing risk for estimating the risks of outcomes. By controlling for the competing risk of death, the Fine and Gray model, which extends the standard univariable and multivariable Cox proportional-hazard regression models, was used to estimate the outcome risks [21].

The differences between the groups were examined using the log-rank test. All statistical analyses were performed using SAS (Version 9.3; SAS Institute Inc., Cary, NC, USA). Results with a two-tailed *p* < 0.05 were considered to indicate a statistically significant relationship.

## 3. Results

### 3.1. Demographic Characteristics

A total of 103,173 patients with ESRD identified from the catastrophic illness database were included in our study (Figure 1). We excluded 42,354 patients diagnosed with POD before undergoing HD. In analysis 1, patients undergoing HD were categorized into the following groups: those with POD who received intensive periodontal therapy (*n* = 6577) and those without a POD diagnosis (*n* = 54,242) between 1 January 1998 and 31 December 2011. After the exclusion process, there were 4149 patients in the treatment and 7169 patients in the control cohort. In analysis 2, the treatment and control cohorts were matched at a 1:1 ratio by the propensity score to reduce selection bias and approximate a randomized trial. Finally, 3613 eligible patients were included in each cohort.

Table 1 presents a comparison of the demographic characteristics of the study patients, both before and after propensity score matching. In the before-matched cohorts, the mean ages were 56.2 ± 12.8 years in the POD treatment group and 60.5 ± 13.8 years in the control group. In the POD treatment group, 55.8% of the patients were women and 72.6% of the patients lived in urbanized areas (level ≥ 2). A Charlson comorbidity index score of 3+, underlying comorbidities, and medication use were more prevalent in the control group than in the POD treatment group.

The distributions of covariates, including age, sex, urbanization level, monthly income, comorbidities, and medication use, were balanced in the two groups after propensity score matching.

### 3.2. Cumulative Incidence and Hazard Ratio of CVD and Mortality

In general, POD treatment was associated with a significantly lower overall CVD risk than that of the untreated group in the multivariate analysis model before propensity score matching(adjusted hazard ratio (aHR) = 0.78, 95% CI = 0.73–0.84, *p* < 0.001; Table 2). Similar results were observed after propensity score matching.

The POD treatment cohorts had lower risks of developing ACS, AMI, stroke (including ischemic stroke and hemorrhagic stroke), CHF, and SCD than those of the untreated group, either before or after matching. POD treatment was also associated with a significantly lower overall mortality risk than that of the untreated group both before and after propensity matching (aHR = 0.50, 95% CI = 0.47–0.54, *p* < 0.001 before matching; aHR = 0.49, 95% CI = 0.45–0.54, *p* < 0.001 after propensity matching).

After the competing risk of death was accounted for, the CVD risk in the treatment group was consistently lower than that in the untreated group in the multivariate analysis model both before and after propensity score matching (adjusted subhazard ratio (aSHR) = 0.77, 95% CI = 0.72–0.82, *p* < 0.001 before matching; aSHR = 0.79, 95% CI = 0.73–0.86, *p* < 0.001 after propensity score matching; Table 3). Similarly, the risks of ACS, AMI, stroke, CHF, and SCD remained significantly decreased in the POD treatment cohort both before and after propensity matching.

**Figure 3:**
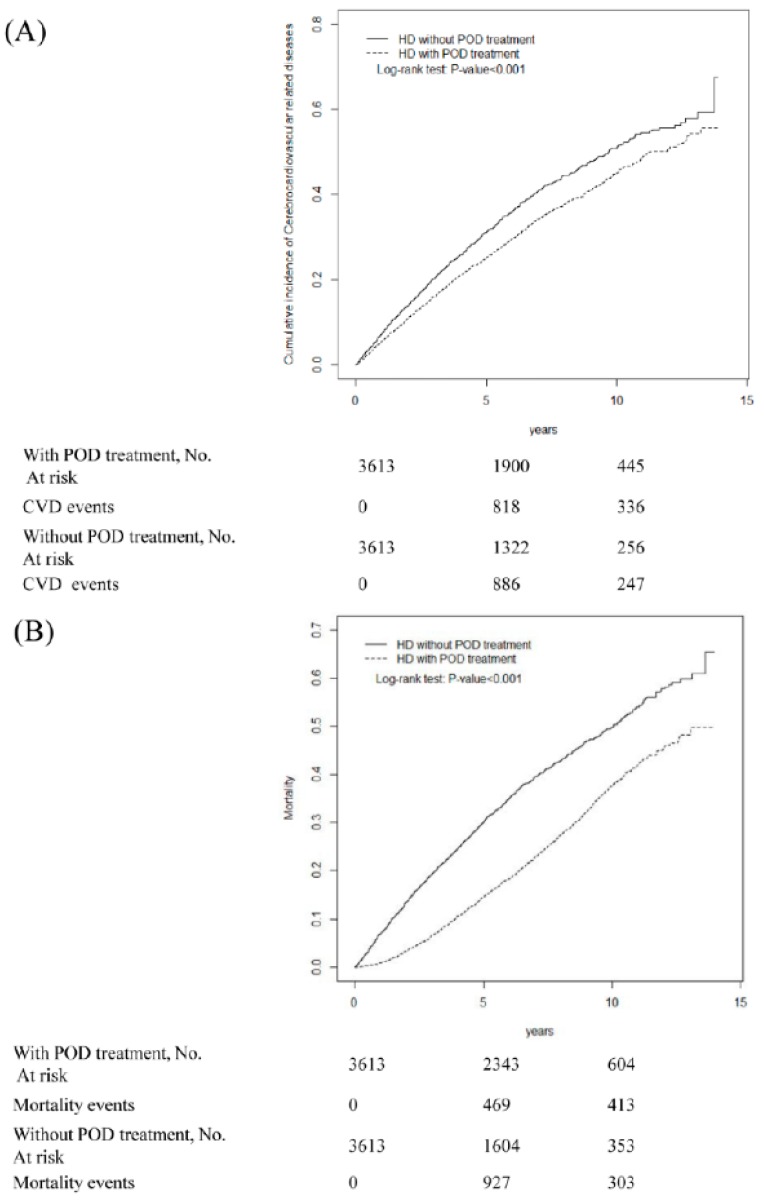
Cumulative incidence of cardiovascular diseases (**A**); and mortality (**B**) for dialysis patients with treatment (dashed line) or controls (solid line). HD: hemodialysis; POD: periodontal disease; CVD: cardiovascular disease.

Figure 3 presents the cumulative incidences of CVD and mortality after 10 years of follow-up in the propensity-matched groups. Compared with the untreated group, the treatment group exhibited significantly lower cumulative incidences of all CVDs (Figure 3A) (log-rank test *p* < 0.001) and mortality (Figure 3B) (log-rank test *p* < 0.001).

### 3.3. Subgroup Analysis

We further stratified the patients according to age, sex, and comorbidities to estimate the risk difference of CVD and mortality (Table 4). The treatment group exhibited lower age-specific relative risks of all CVDs. Both men and women in the treatment group had significantly lower risks of CVDs compared with those in the control group. The patients with comorbidity in the treatment group had lower risks of CVDs compared with those in the control group, and the significant interaction term between treatment and comorbidities was observed (*p* for interaction = 0.04). For risk stratifications of overall mortality between the groups, the risks of mortality were significantly lower in the treatment group compared with those of the control group, regardless of age, sex, or comorbidity status. An interaction between treatment and comorbidities was also observed (*p* for interaction = 0.04) for mortality analysis.

In addition, we conducted sensitivity analyses to validate our findings (Table 5). We defined the effects of treatment responsiveness according to the frequency of clinic visitation for intensive periodontal treatment. Compared with the untreated group, the risks of CVDs were increased in patients with POD who received fewer than one course of treatment, but these risks were reduced significantly in patients with more than 2 treatment courses, with an aHR of 0.53 (95% CI = 0.48–0.59, *p* < 0.001). Significant risk reductions of ACS, AMI, stroke, CHF, and SCD in the treatment group were also observed in patients who underwent more courses of intensive treatment, compared with those in the untreated group. The risk of overall mortality was reduced in the treatment cohort regardless of the treatment frequency, compared with that in the untreated group.

## 4. Discussion

Our study is the first to identify an independent association between intensive POD therapy and new-onset major CVDs in patients on HD. Patients on HD with POD therapy showed a 22% risk reduction in all CVDs compared with patients on HD without POD treatment, after competing risk model analysis.

### 4.1. Cardiovascular Diseases

Bacteria colonizing periodontal pockets and those found in dental plaques function as POD pathogens. They elicit local inflammatory responses and invade circulation to induce systemic inflammatory responses. Markers of systemic inflammation, including C-reactive protein and interleukin 6, are surrogate markers of atherosclerotic vascular disease and are increased in patients with periodontitis [22]. A strong association was observed between POD and the risks of atherosclerosis vascular disease, ACS, and stroke in the general population [23]. Endothelial dysfunction and atherosclerosis caused by POD were postulated to be mediated by direct infection, bacteremia, or molecular mimicry [24].

Cardiovascular events caused by POD may at least be partially mediated by inflammation and endothelial dysfunction. Most studies have reported improved endothelial function and reduced inflammation among patients with POD who underwent periodontal interventions [25,26,27]. These findings support our hypothesis that POD is a marker of a susceptible immune system or might directly affect cardiovascular and mortality risks, which may be modified by POD treatment.

One study reports the occurrence of short-term systemic inflammation and endothelial dysfunction after intensive periodontal treatment [26]; another study suggested that dental procedures were correlated with short-term increased risks of vascular events [23]. These observations further support the assertion that bacterial translocation and procedure-related inflammation after dental procedures exert negatively effect on endothelial function.

No consensus has been reached regarding the role of POD interventions in the primary or secondary prevention of CVDs in patients with POD. In addition, few data on deaths (all-cause or CVD mortality) are available [28]. As we demonstrated that intensive POD therapy plays a role in the primary prevention of CVDs and has benefits for survival, we recommend that the diagnosis and management of POD not be overlooked in high-risk patients on dialysis.

### 4.2. All-Cause Mortality

The presence of moderate to severe POD adversely affected the survival of patients with ESRD [15]. The association between POD and mortality in the dialysis population was confirmed [13]. In our previous cohort study, intensive POD treatment was associated with reduced risks of overall infectious diseases in patients on HD [19]. Thus, the benefits of POD treatment in reducing the risks of CVDs and infectious diseases may contribute to favorable survival outcomes. The survival benefits also increased with the frequency of POD treatment.

In addition to factors related to dialysis, risk factors such as comorbid conditions and malnutrition affect patient survival [29]. POD is associated with malnutrition in patients on HD [30]. The oral health status can affect eating habits and thus the nutrition status [31]. Improvement in dental hygiene after POD treatment may have a clinical impact on nutrition; however, further research is necessary to determine whether this effect translates into survival benefits for patients on dialysis.

### 4.3. Congestive Heart Failure

Approximately 80% of patients on HD had cardiac diseases including ischemic heart disease, CHF, and arrhythmia. Among all CVDs in our study, the incidence rate of CHF was the highest, being at 33.0 per 1000 person-years in the comparison cohort. Management of CHF in patients on HD includes treating underlying diseases, optimal volume control, and pharmacologic therapy including the use of beta-blockers and ACEis [10]. Our study demonstrated that POD treatment was associated with a 29% lower risk of CHF in patients on dialysis. The beneficial effect of POD treatment on endothelial function and systemic inflammation may translate into a reduction in CHF events in patients on dialysis.

### 4.4. Acute Coronary Syndromeand Acute Myocardial Infarction

Approximately 20% of cardiac deaths in patients on dialysis were attributed to AMI [32]. The increased prevalence of periodontal pathogens was associated with an increased risk of MI in the general population [33]. A study from Sweden in 2016 also demonstrated an association between periodontitis and the risk of a first myocardial infarction [34]. Few studies have investigated the effect of POD treatment on ACS. A current ongoing trial will provide new evidence regarding the effect of POD treatment on the risk of CVD recurrence in patients with stable coronary artery disease [35]. Additional studies are necessary to evaluate the relationship between improved cardiovascular biomarkers and clinical endpoints in patients after POD treatment.

### 4.5. Stroke

Our results showed a 28% risk reduction in overall stroke in the treatment cohort, largely resulting from the reduced incidence of ischemic stroke. Despite evidence indicating that POD is associated with ischemic stroke [36], little is known regarding the association between POD and hemorrhagic stroke. Only one case–control study demonstrated an association between periodontitis and hemorrhagic stroke [37]. Future prospective studies investigating biologic parameters are necessary to establish causality.

## 5. Limitations

The strength of our study is that it is the first to demonstrate that POD therapy might be beneficial in the primary prevention of CVDs and might improve survival in the HD population. However, our results should be interpreted with caution in consideration of the inherent limitations in a retrospective, observational study.

First, our findings were obtained by analyzing population-based data and could be generalized to the hemodialysis population. Potential problems in generalizing the results to the overall ESRD population could be the variations in the POD incidence among different races and dialysis modalities, such as peritoneal dialysis.

Second, potential risk factors are shared by CVDs and POD, such as smoking, alcohol abuse, and obesity. This information is not available in the NHIRD; hence, we could not adjust for these health-related factors. However, potential effect modifiers such as income and urbanization level were included in the analysis. Evidence from a study cohort including nonsmokers supports the association between POD and CVDs [38], suggesting an independent effect of POD on CVDs.

Finally, the number of POD patients who received no POD treatment in our database was only 132, which is too low to be a control group. This unbalanced distribution could be explained by the link between diagnosis and treatment caused by the copayment rule of the NHI program. We were uncertain about the POD status of the comparison cohort. POD diagnosis codes might have been absent for patients on HD because the patients did not have POD, or because they had POD that was underdiagnosed. Oral diseases may be underdiagnosed in patients on HD because of the low rate of public dental service utilization [39,40]. As observed in our study, the high prevalence of comorbidities might prevent patients from seeking dental care. Therefore, we selected HD patients without POD diagnoses from the database as the comparison cohort. The potential bias resulting from the control group selection does not, however, weaken the positive results of the study.

## 6. Conclusions

POD treatment was associated with a significant decrease in all-cause mortality and CVD events in HD patients. POD may be a potentially modifiable risk factor for CVDs in patients on HD. Further clinical investigation may be necessary to conclude whether periodontal intervention could be a potential prevention measure for CVDs in HD patients.

## Figures and Tables

**Table 1:** Demographic status and comorbidity at baseline in hemodialysis patients with and without periodontal disease treatment.

Variables	Before Matching			Propensity Score Matched		
	Controls (*n* = 7169) *n* (%)	Treatment (*n* = 4149) *n* (%)	Standardized Differences	Controls (*n* = 3613) *n* (%)	Treatment (*n* = 3613) *n* (%)	Standardized Differences
Age (year), mean ± SD	60.5 (13.8)	56.2 (12.8)	0.32	57.5 (13.8)	57.4 (12.5)	0.003
Follow-up time (year) for CVD, mean ± SD	3.58 (2.83)	6.00 (3.60)	0.75	4.34 (3.25)	5.60 (3.37)	0.38
Follow-up time (year) for mortality, mean ± SD	4.10 (2.95)	7.02 (3.41)	0.92	4.97 (3.34)	6.56 (3.22)	0.48
Gender						
Women	3661 (51.1)	2314 (55.8)	0.09	1941 (53.7)	1966 (54.4)	0.014
Men	3508 (48.9)	1835 (44.2)	0.09	1672 (46.3)	1647 (45.6)	0.014
Dialysis duration (years)	3.17 (2.73)	2.36 (2.21)	0.33	2.52 (2.23)	2.54 (2.28)	0.009
Urbanization level ^†^						
1	1626 (22.7)	1138 (27.4)	0.09	917 (25.4)	929 (25.7)	0.014
2	2105 (29.4)	1305 (31.5)	0.09	1114 (30.8)	1125 (31.1)	0.014
3	1268 (17.7)	732 (17.6)	0.09	645 (17.9)	642 (17.8)	0.014
4+	2170 (30.3)	974 (23.5)	0.09	937 (25.9)	917 (25.4)	0.014
Monthly income (NTD) *						
<15,000	1983 (27.7)	1051 (25.3)	0.09	947 (26.2)	955 (26.4)	0.014
15,000–19,999	3886 (54.2)	2093 (50.5)	0.09	1875 (51.9)	1850 (51.2)	0.014
≥20,000	1300 (18.1)	1005 (24.2)	0.09	791 (21.9)	808 (22.4)	0.014
Charlson comorbidity index						
0	605 (8.44)	443 (10.7)	0.09	351 (9.71)	349 (9.66)	0.014
1	91 (1.27)	34 (0.82)	0.09	330 (0.83)	31 (0.86)	0.014
2	3157 (44.0)	2181 (52.6)	0.09	1835 (50.8)	1831 (50.7)	0.014
3+	3316 (46.3)	1491 (35.9)	0.09	1397 (38.7)	1402 (38.8)	0.014
Comorbidity						
COPD	967 (13.5)	476 (11.5)	0.06	436 (12.1)	421 (11.7)	0.013
Hyperlipidemia	2294 (32.0)	1225 (29.5)	0.05	1092 (30.2)	1100 (30.5)	0.005
Diabetes	2406 (33.6)	965 (23.3)	0.23	947 (26.2)	932 (25.8)	0.009
Hypertension	6025 (84.0)	3432 (82.7)	0.04	2992 (82.8)	2991 (82.8)	0.001
PAOD	519 (7.24)	220 (5.30)	0.08	206 (5.70)	207 (5.73)	0.001
Cancer	516 (7.20)	226 (5.45)	0.07	201 (5.56)	209 (5.78)	0.01
Lower extremity amputation	226 (3.15)	45 (1.08)	0.14	49 (1.36)	45 (1.25)	0.01
Medication						
Anti-platelet agents	3800 (53.0)	1892 (45.6)	0.15	1676 (46.4)	1747 (48.4)	0.04
ACEis/ARBs	5585 (77.9)	3056 (73.4)	0.10	2684 (74.3)	2718 (75.2)	0.02
Statins	2173 (30.3)	1009 (24.3)	0.14	965 (26.7)	928 (25.7)	0.02
Calcium channel blockers	5833 (81.4)	3156 (76.1)	0.13	2832 (78.4)	2797 (77.4)	0.02
Beta-blockers	4656 (65.0)	2545 (61.3)	0.08	2230 (61.7)	2270 (62.8)	0.02

ACEis: angiotensin-converting enzyme (ACE) inhibitors; ARBs: angiotensin II receptor blockers; COPD: chronic obstructive pulmonary disease; CVD: cardiovascular disease; PAOD: peripheral arterial occlusive disease. ^†^ The urbanization level was categorized by the population density of the residential area into four levels, with level 1 as the most urbanized and level 4 as the least urbanized. * New Taiwan Dollars (NTD; US dollar 1.0 is equivalent to approximately NTD30). SD: standard deviation.

**Table 2:** Outcomes of periodontal disease (POD) treatment and control groups, determined using the matched Cox proportional hazard model.

Outcome	Controls			Treatment			Crude HR (95% CI)	Adjusted HR (95% CI) ^‡^
	Event	PY	Rate ^#^	Event	PY	Rate		
**Before match**								
CVDs	2173	25,680	84.6	1366	24,887	54.9	0.66 (0.62, 0.71) ***	0.78 (0.73, 0.84) ***
ACS	642	28,336	22.7	466	27,720	16.8	0.73 (0.65, 0.83) ***	0.87 (0.77, 0.99) *
AMI	326	28,989	11.3	196	28,737	6.82	0.58 (0.49, 0.70) ***	0.72 (0.59, 0.86) ***
Stroke	1011	27,831	36.3	600	27,477	21.8	0.61 (0.55, 0.68) ***	0.72 (0.65, 0.81) ***
Ischemic stroke	732	-	26.3	461	27,477	16.8	0.66 (0.59, 0.74) ***	0.82 (0.72, 0.92) **
Hemorrhagic stroke	279	-	10.0	139	-	5.06	0.49 (0.40, 0.61) ***	0.53 (0.43, 0.65) ***
CHF	911	27,616	33.0	683	26,992	25.3	0.77 (0.70, 0.86) ***	0.89 (0.80, 0.99) *
SCD	155	29,328	5.29	74	29,071	2.55	0.45 (0.34, 0.59) ***	0.55 (0.41, 0.74) ***
Mortality	2417	29,385	82.3	1055	29,113	36.2	0.42 (0.39, 0.45) ***	0.50 (0.47,0.54) ***
**Propensity Score Matched**								
CVDs	1156	15,684	73.7	1197	20,215	59.2	0.81 (0.74, 0.87) ***	0.75 (0.69, 0.81) ***
ACS	339	17,344	19.6	410	22,533	18.2	0.92 (0.80, 1.06)	0.85 (0.74, 0.99) *
AMI	169	17,752	9.52	181	23,373	7.74	0.79 (0.64, 0.97) *	0.72 (0.58, 0.89) **
Stroke	553	17,061	32.4	518	22,315	23.2	0.72 (0.64, 0.81) ***	0.67 (0.60, 0.76) ***
Ischemic stroke	369	-	21.6	402	-	18.0	0.84 (0.73, 0.97) *	0.78 (0.68, 0.90) ***
Hemorrhagic stroke	184	-	10.8	116	-	5.20	0.48 (0.38, 0.61) ***	0.47 (0.37, 0.59) ***
CHF	500	16,817	29.7	590	22,013	26.8	0.90 (0.80, 1.01)	0.83 (0.73, 0.93) **
SCD	66	17,939	3.68	69	23,661	2.92	0.75 (0.54, 1.06)	0.68 (0.48, 0.95) *
Mortality	1274	17,972	70.9	945	23,697	39.9	0.55 (0.50, 0.59) ***	0.49 (0.45, 0.54) ***

Rate ^#^ incidence rate per 1000 person-years; PY: person-years, ^‡^ Multivariable analysis including age, gender, urbanization level, monthly income, Charlson comorbidity index, comorbidities (chronic obstructive pulmonary disease (COPD), hyperlipidemia, diabetes, hypertension, peripheral arterial occlusive disease (PAOD), and lower extremity amputation), and medications (anti-platelet agents, angiotensin-converting enzyme (ACE) inhibitors (ACEis)/angiotensin II receptor blockers (ARBs), statins, calcium channel blockers, and beta-blockers). * *p* < 0.05, ** *p* < 0.01, *** *p* < 0.001. CVDs: cardiovascular diseases; ACS: acute coronary syndrome; AMI: acute myocardial infarction; CHF: congestive heart failure; SCD: sudden cardiac death; HR: hazard ratio; CI: confidence interval.

**Table 3:** Competing risk (death) model-measured subhazard ratios (SHRs) of outcomes with and without propensity score matching between groups.

Variables	Before Matching		Propensity Score Matched	
	Controls (*n* = 7169)	Treatment (*n* = 4149)	Controls (*n* = 3613)	Treatment (*n* = 3613)
CVDs				
Crude SHR (95% CI)	1 (Ref)	0.74 (0.69,0.80) ***	1 (Ref)	0.78 (0.72, 0.84) ***
Adjusted SHR ^a^ (95% CI)	1 (Ref)	0.77 (0.72, 0.82) ***	1 (Ref)	0.79 (0.73, 0.86) ***
ACS				
Crude SHR (95% CI)	1 (Ref)	0.74 (0.66, 0.83) ***	1 (Ref)	0.84 (0.73, 0.97) *
Adjusted SHR ^a^ (95% CI)	1 (Ref)	0.81 (0.72, 0.91) ***	1 (Ref)	0.83 (0.72, 0.95) **
AMI				
Crude SHR (95% CI)	1 (Ref)	0.54 (0.46, 0.64) ***	1 (Ref)	0.68 (0.56, 0.84) ***
Adjusted SHR ^a^ (95% CI)	1 (Ref)	0.63 (0.53, 0.76) ***	1 (Ref)	0.68 (0.56, 0.84) ***
Stroke				
Crude SHR (95% CI)	1 (Ref)	0.62 (0.56, 0.68) ***	1 (Ref)	0.66 (0.58, 1.74) ***
Adjusted SHR ^a^ (95% CI)	1 (Ref)	0.58 (0.53, 0.65) ***	1 (Ref)	0.72 (0.63, 0.81) ***
Ischemic stroke				
Crude SHR (95% CI)	1 (Ref)	0.62 (0.56, 0.70) ***	1 (Ref)	0.71 (0.62, 0.82) ***
Adjusted SHR ^a^ (95% CI)	1 (Ref)	0.63 (0.56, 0.71) ***	1 (Ref)	0.81 (0.70, 0.93) **
Hemorrhagic stroke				
Crude SHR (95% CI)	1 (Ref)	0.48 (0.39, 0.59) ***	1 (Ref)	0.46 (0.37, 0.58) ***
Adjusted SHR ^a^ (95% CI)	1 (Ref)	0.42 (0.34, 0.52) ***	1 (Ref)	0.50 (0.40, 0.64) ***
CHF				
Crude SHR (95% CI)	1 (Ref)	0.81 (0.73, 0.89) ***	1 (Ref)	0.83 (0.74, 0.93) **
Adjusted SHR ^a^ (95% CI)	1 (Ref)	0.85 (0.77, 0.94) **	1 (Ref)	0.81 (0.72, 0.92) ***
SCD				
Crude SHR (95% CI)	1 (Ref)	0.40 (0.30, 0.52) ***	1 (Ref)	0.60 (0.44, 0.83) **
Adjusted SHR ^a^ (95% CI)	1 (Ref)	0.52 (0.39, 0.68) ***	1 (Ref)	0.64 (0.46, 0.89) **

Ref: reference group. ^a^ Cox proportional hazard regression, adjusted for age, gender, urbanization level, monthly income, Charlson comorbidity index, comorbidities (chronic obstructive pulmonary disease (COPD), hyperlipidemia, diabetes, hypertension, peripheral arterial occlusive disease (PAOD), and lower extremity amputation) and medications (anti-platelet agents, angiotensin-converting enzyme (ACE) inhibitors (ACEis)/angiotensin II receptor blockers (ARBs), statins, calcium channel blockers, and beta-blockers). CVDs: cardiovascular diseases; ACS: acute coronary syndrome; AMI: acute myocardial infarction; CHF: congestive heart failure; SCD: sudden cardiac death. * *p* < 0.05, ** *p* < 0.01, *** *p* < 0.001. SHR: subhazard ratio; CI: confidence interval.

**Table 4:** Matched Cox proportional hazards model measured hazard ratios (HRs) and 95% confidence intervals (CIs) of outcomes of patients with periodontal disease after treatment and patient without POD by age, gender, and comorbidity.

Variables	Propensity Score Matched						Crude HR (95% CI)	Adjusted HR (95% CI)
	Controls			Periodontal Disease after Treatment				
	Event	PY	Rate ^†^	Event	PY	Rate ^†^		
CVDs ^1^								
Age, years								
≤49	252	6323	39.9	204	6790	29.6	0.75 (0.62, 0.90) **	0.70 (0.58, 0.84) ***
50–64	451	5787	77.9	543	8428	64.4	0.81 (0.72, 0.92) **	0.82 (0.72, 0.93) **
≥65	453	3574	126.8	453	4997	90.7	0.70 (0.62, 0.80) ***	0.68 (0.60, 0.78) ***
*p* for interaction								0.81
Gender								
Women	616	8757	70.3	618	11,512	53.7	0.77 (0.69, 0.86) ***	0.73 (0.65, 0.82) ***
Men	540	6927	78.0	579	8703	66.5	0.85 (0.76, 0.96) **	0.79 (0.70, 0.89) ***
*p* for interaction								0.81
Comorbidity ^§^								
No	98	2434	40.3	102	3238	31.5	0.77 (0.58, 1.02)	0.76 (0.57, 1.00)
Yes	1098	13,250	79.9	1095	16,977	64.5	0.81 (0.74, 0.88) ***	0.76 (0.70, 0.83) ***
*p* for interaction								0.04
ACS ^2^								
Age, years								
≤49	72	6749	10.7	66	7245	9.11	0.85 (0.61, 1.18)	0.79 (0.56, 1.11)
50–64	132	6457	20.4	193	9561	20.2	0.96 (0.77, 1.20)	0.99 (0.79, 1.23)
≥65	135	4137	32.6	151	5727	26.4	0.78 (0.62, 0.99) *	0.74 (0.59, 0.94) *
*p* for interaction								0.88
Gender								
Women	164	9708	16.9	203	12,829	15.8	0.93 (0.76, 1.15)	0.86 (0.70, 1.06)
Men	175	7636	22.9	207	9704	21.3	0.91 (0.75, 1.12)	0.84 (0.69, 1.03)
*p* for interaction								0.97
Comorbidity ^§^								
No	26	2577	10.1	22	3503	6.28	0.61 (0.34, 1.07)	0.56 (0.31, 0.99) *
Yes	313	14,767	21.2	388	19,030	20.4	0.95 (0.82, 1.10)	0.90 (0.78, 1.05)
*p* for interaction								0.15
AMI ^2^								
Age, years								
≤49	31	6875	4.51	27	7395	3.65	0.79 (0.47, 1.32)	0.67 (0.39, 1.13)
50–64	67	6623	10.1	88	9932	8.86	0.84 (0.61, 1.15)	0.88 (0.64, 1.21)
≥65	71	4254	10.7	66	6046	10.9	0.61 (0.44, 0.86) **	0.59 (0.42, 0.84) **
*p* for interaction								0.73
Gender								
Women	84	9912	8.47	77	13,262	5.81	0.67 (0.49, 0.91) *	0.60 (0.43, 0.82) **
Men	85	7840	10.8	104	10,110	10.3	0.91 (0.68, 1.22)	0.89 (0.66, 1.19)
*p* for interaction								0.13
Comorbidity ^§^								
No	14	2606	5.37	10	3544	2.82	0.51 (0.22, 1.14)	0.45 (0.19, 1.04)
Yes	155	15,146	10.2	171	19,829	8.62	0.81 (0.65, 1.01)	0.75 (0.61, 0.94) *
*p* for interaction								0.28
Stroke ^3^								
Age, years								
≤49	117	6739	17.4	79	7229	10.9	0.63 (0.47, 0.84) **	0.58 (0.44, 0.78) ***
50–64	215	6332	34.0	242	9420	25.7	0.75 (0.62, 0.90) **	0.75 (0.62, 0.90) **
≥65	221	3990	55.4	197	5666	34.8	0.62 (0.51, 0.76) ***	0.62 (0.51, 0.76) ***
*p* for interaction								0.93
Gender								
Women	305	9512	32.1	275	12,589	21.8	0.68 (0.58, 0.81) ***	0.65 (0.55, 0.77) ***
Men	248	7548	32.9	243	9726	25.0	0.77 (0.64, 0.91) **	0.71 (0.60, 0.85) ***
*p* for interaction								0.36
Comorbidity ^§^								
No	38	2548	14.9	43	3432	12.5	0.82 (0.53, 1.27)	0.79 (0.51, 1.23)
Yes	515	14,513	35.5	475	18,883	25.2	0.71 (0.63, 0.81) ***	0.67 (0.59, 0.76) ***
*p* for interaction								0.47
CHF ^4^								
Age, years								
≤49	99	6619	15.0	99	7144	13.9	0.92 (0.70, 1.22)	0.87 (0.65, 1.15)
50–64	195	6215	31.4	262	9301	28.2	0.87 (0.72, 1.05)	0.86 (0.72, 1.04)
≥65	206	3983	51.7	229	5568	41.1	0.78 (0.65, 0.95) *	0.74 (0.61, 0.90) **
*p* for interaction								0.69
Gender								
Women	270	9363	28.8	312	12,449	25.1	0.87 (0.74, 1.03)	0.83 (0.70, 0.97) *
Men	230	7454	30.9	278	9564	29.1	0.92 (0.78, 1.10)	0.84 (0.70, 0.99) *
*p* for interaction								0.50
Comorbidity ^§^								
No	42	2517	16.7	56	3383	16.6	0.97 (0.65, 1.45)	0.92 (0.61, 1.37)
Yes	458	14,300	32.0	534	18,630	28.7	0.89 (0.78, 1.01)	0.82 (0.72, 0.92) **
*p* for interaction								0.64
SCD ^5^								
Age, years								
≤49	11	6924	1.59	10	7459	1.34	0.80 (0.34, 1.88)	0.74 (0.31, 1.77)
50–64	23	6687	3.44	31	10,101	3.07	0.85 (0.49, 1.45)	0.83 (0.48, 1.44)
≥65	32	4329	7.39	28	6101	4.59	0.56 (0.33, 0.93) *	0.55 (0.33, 0.92) *
*p* for interaction								0.36
Gender								
Women	39	10,010	3.90	35	13,366	2.62	0.64 (0.40, 1.01)	0.59 (0.37, 0.93) *
Men	27	7930	3.40	34	10,295	3.30	0.92 (0.55, 1.53)	0.86 (0.51, 1.44)
*p* for interaction								0.31
Comorbidity ^§^								
No	8	2610	3.06	8	3564	2.24	0.70 (0.26, 1.87)	0.75 (0.27, 2.09)
Yes	58	15,329	3.78	61	20,097	3.04	0.76 (0.53, 1.09)	0.69 (0.48, 0.99) *
*p* for interaction								0.86
Mortality ^6^								
Age, years								
≤49	215	6933	31.0	129	7460	17.3	0.55 (0.44, 0.69) ***	0.50 (0.40, 0.62) ***
50–64	485	6694	72.5	383	10,125	37.8	0.49 (0.43, 0.56) ***	0.48 (0.42, 0.55) ***
≥65	574	4345	132.1	433	6112	70.9	0.50 (0.44, 0.57) ***	0.48 (0.42, 0.54) ***
*p* for interaction								0.81
Gender								
Women	631	10,030	62.9	468	13,382	35.0	0.54 (0.48, 0.61) ***	0.52 (0.46, 0.59) ***
Men	643	7943	81.0	477	10,315	46.2	0.55 (0.49, 0.62) ***	0.47 (0.42, 0.53) ***
*p* for interaction								0.81
Comorbidity ^§^								
No	160	2615	61.2	95	3564	26.7	0.42 (0.33, 0.54) ***	0.39 (0.30, 0.51) ***
Yes	1114	15,357	72.5	850	20,133	42.2	0.56 (0.51, 0.62) ***	0.51 (0.46, 0.55) ***
*p* for interaction								0.04

CVDs: cardiovascular diseases; CI: confidence interval; HR: relative hazard ratio; PY: person-years; ACS: acute coronary syndrome; AMI: acute myocardial infarction; CHF: congestive heart failure; SCD: sudden cardiac death; ^†^ Rate: Incidence rate per 1000 person-years, Comorbidity ^§^ Patients with comorbidities (including chronic obstructive pulmonary disease (COPD), hyperlipidemia, diabetes, hypertension, peripheral arterial occlusive disease (PAOD), cancer, and lower extremity amputation) were classified as the comorbidity group. * *p* < 0.05, ** *p* < 0.01, *** *p* < 0.001, ^1^ Adjusted HR was calculated by Cox proportional hazard regression and adjusted for age, gender, monthly income, Charlson comorbidity index, comorbidities (COPD, hyperlipidemia, diabetes, hypertension, PAOD, and low extremity amputation), and medication (anti-platelet agents, angiotensin-converting enzyme (ACE) inhibitors (ACEis)/angiotensin II receptor blockers (ARBs), statins, calcium channel blockers, and beta-blockers). ^2^ Adjusted HR was calculated by Cox proportional hazard regression and adjusted for age, gender, monthly income, Charlson comorbidity index, comorbidities (hyperlipidemia, diabetes, hypertension, PAOD, and lower extremity amputation), and medication (anti-platelet agents, ACEi/ARB, statins, calcium channel blockers, and beta-blockers). ^3^ Adjusted HR was calculated by Cox proportional hazard regression and adjusted for age, monthly income, Charlson comorbidity index, comorbidities (COPD, hyperlipidemia, diabetes, hypertension, PAOD, and lower extremity amputation),and medication (anti-platelet agents, ACEis/ARBs, statins, calcium channel blockers, and beta-blockers). ^4^ Adjusted HR was calculated by Cox proportional hazard regression and adjusted for age, gender, urbanization level, monthly income, Charlson comorbidity index, comorbidities (COPD, hyperlipidemia, diabetes, hypertension, and PAOD), and medication (anti-platelet agents, ACEis/ARBs, statins, calcium channel blockers, and beta-blockers). ^5^ Adjusted HR was calculated by Cox proportional hazard regression and adjusted for age, urbanization level, Charlson comorbidity index, comorbidities (hyperlipidemia and diabetes), and medication (beta-blockers). ^6^ Adjusted HR was calculated by Cox proportional hazard regression and adjusted for age, gender, urbanization level, monthly income, Charlson comorbidity index, comorbidities (COPD, hyperlipidemia, diabetes, hypertension, PAOD, cancer and lower extremity amputation), and medication (anti-platelet agents, ACEi/ARB, statins, calcium channel blockers, and beta-blockers).

**Table 5:** Matched Cox proportional hazards model measured hazard ratio (HR) and 95% confidence interval (CIs) between outcome events and the frequency of clinic visit for intensive periodontal disease treatment.

Effects of Intensive Periodontal Disease Treatment on Major Cardiovascular Events	Hazard Ratio (95% CI)		
	Number of Events	Rate ^#^	Adjusted HR ^†^
Frequency of treatment of periodontal disease			
CVDs ^1^			
Controls	1156	73.7	1 (Reference)
Frequency of treatment of periodontal disease			
≤1	587	120.5	1.29 (1.17, 1.43) ***
≥2	610	39.8	0.53 (0.48, 0.59) ***
*p* for trend			<0.001
ACS ^2^			
Controls	339	19.6	1 (Reference)
Frequency of treatment of periodontal disease			
≤1	210	33.6	1.33 (1.11, 1.58) **
≥2	200	12.3	0.62 (0.52, 0.74) ***
*p* for trend			<0.001
AMI ^2^			
Controls	169	9.52	1 (Reference)
Frequency of treatment of periodontal disease			
≤1	93	13.7	1.05 (0.81, 1.36)
≥2	88	5.30	0.53 (0.41, 0.69) ***
*p* for trend			<0.001
Stroke ^3^			
Controls	553	32.4	1 (Reference)
Frequency of treatment of periodontal disease			
≤1	262	42.6	1.04 (0.89, 1.20)
≥2	256	15.8	0.50 (0.43, 0.58) ***
*p* for trend			<0.001
CHF ^4^			
Controls	500	29.7	1 (Reference)
Frequency of treatment of periodontal disease			
≤1	313	52.3	1.39 (1.20, 1.60) ***
≥2	277	17.3	0.57 (0.49, 0.66) ***
*p* for trend			<0.001
Sudden cardiac death ^5^			
Controls	66	3.68	1 (Reference)
Frequency of treatment of periodontal disease			
≤1	25	3.59	0.70 (0.44, 1.11)
≥2	44	2.64	0.66 (0.45, 0.98) *
*p* for trend			0.004
Mortality ^6^			
Controls	1274	70.9	
Frequency of treatment of periodontal disease			1 (Reference)
≤1	369	52.8	0.56 (0.50, 0.63) ***
≥2	576	34.5	0.46 (0.42, 0.51) ***
*p* for trend			<0.001

CVDs: cardiovascular diseases; ACS: acute coronary syndrome; AMI: acute myocardial infarction; CHF: congestive heart failure; Rate ^#^ incidence rate, per 1000 person-years; **^†^** adjusted HR by Cox proportional hazard regression, adjusted for age, gender, urbanization level, monthly income, Charlson comorbidity index, comorbidities (chronic obstructive pulmonary disease (COPD), hyperlipidemia, diabetes, hypertension, peripheral arterial occlusive disease (PAOD), and lower extremity amputation), and medications (anti-platelet agents, angiotensin-converting enzyme (ACE) inhibitors (ACEis)/angiotensin II receptor blockers (ARBs), statins, calcium channel blockers, and beta-blockers). ^1^ Adjusted HR was calculated by Cox proportional hazard regression and adjusted for age, gender, monthly income, Charlson comorbidity index, comorbidities (COPD, hyperlipidemia, diabetes, hypertension, PAOD, and low extremity amputation), and medication (anti-platelet agents, ACEis/ARBs, statins, calcium channel blockers, and beta-blockers). ^2^ Adjusted HR was calculated by Cox proportional hazard regression and adjusted for age, gender, monthly income, Charlson comorbidity index, comorbidities (hyperlipidemia, diabetes, hypertension, PAOD, and low extremity amputation), and medication (anti-platelet agents, ACEis/ARBs, statins, calcium channel blockers, and beta-blockers). ^3^ Adjusted HR was calculated by Cox proportional hazard regression and adjusted for age, monthly income, Charlson comorbidity index, comorbidities (COPD, hyperlipidemia, diabetes, hypertension, PAOD, and lower extremity amputation), and medication (anti-platelet agents, ACEis/ARBs, statins, calcium channel blockers, and beta-blockers). ^4^ Adjusted HR was calculated by Cox proportional hazard regression and adjusted for age, gender, urbanization level, monthly income, Charlson comorbidity index, comorbidities (COPD, hyperlipidemia, diabetes, hypertension, and PAOD), and medication (anti-platelet agents, ACEis/ARBs, statins, calcium channel blockers, and beta-blockers). ^5^ Adjusted HR was calculated by Cox proportional hazard regression and adjusted for age, urbanization level, Charlson comorbidity index, comorbidities (hyperlipidemia and diabetes), and medication (beta-blockers). ^6^ Adjusted HR was calculated by Cox proportional hazard regression and adjusted for age, gender, urbanization level, monthly income, Charlson comorbidity index, comorbidities (COPD, hyperlipidemia, diabetes, hypertension, PAOD, cancer, and low extremity amputation), and medication (anti-platelet agents, ACEis/ARBs, statins, calcium channel blockers, and beta-blockers). * *p* < 0.05; ** *p* < 0.01; *** *p* < 0.001.
